# Editorial: Preventing cardiovascular complications of type 2 diabetes

**DOI:** 10.3389/fendo.2024.1473603

**Published:** 2024-08-09

**Authors:** Maria Pompea Antonia Baldassarre, Teresa Paolucci, Kyoungmin Park, Caterina Pipino

**Affiliations:** ^1^ Department of Medicine and Aging Sciences, G. d’Annunzio University, Chieti, Italy; ^2^ Center for Advanced Studies and Technology (CAST), G. d’Annunzio University, Chieti, Italy; ^3^ Department of Medical, Oral and Biotechnological Sciences, Behavioral Imaging and Neural Dynamics (BIND) Center, Physical and Rehabilitation Medicine, G. d’Annunzio University, Chieti, Italy; ^4^ Research Division, Joslin Diabetes Center, Department of Medicine, Harvard Medical School, Boston, MA, United States; ^5^ Department of Medical, Oral and Biotechnological Sciences, G. d’Annunzio University, Chieti, Italy

**Keywords:** cardiovascular complications, type 2 diabetes, lifestyle modifications, medical management, community support, exercise

The global prevalence of type 2 diabetes (T2D) continues to rise at an alarming rate, bringing with it a myriad of health complications, the most critical of which are cardiovascular diseases (CVD) (1). As we grapple with this public health challenge, it becomes imperative to address the prevention of cardiovascular complications in individuals with T2D. These complications, ranging from myocardial infarction, stroke, heart failure, risk of cardiac rupture (Zu et al.) to peripheral artery disease, pose significant risks not only to the quality of life but also to overall mortality. Therefore, a comprehensive approach involving lifestyle changes, medical management, and community support is essential for mitigating these risks.

## Unveiling hidden risks: early biomarkers for diabetes complications

Recent advances in biomarker research have opened new avenues for early detection and prevention of diabetes-related complications, potentially revolutionizing patient care and outcomes (2). The identification of useful biomarkers is crucial, as highlighted in this Research Topic. Chang et al. developed a novel nomogram with a simple graphical format for predicting the risk of cardiometabolic diseases which can be used by primary healthcare providers for predicting CVD and preventing their progression. Another intriguing potential approach for early diagnosis is the one explored by Tusongtuoheti et al., who employed interpretable machine learning models to predict the risk of subclinical atherosclerosis in patients with T2D in China. Interestingly, age, albumin, total protein, total cholesterol, and serum creatinine have been identified as the top five contributing variables in this predictive model.

In the study by Cui et al., two datasets were integrated to identify differentially expressed genes (DEGs) between controls and type 2 diabetic cardiomyopathy cases. The team analyzed immune cell infiltration, constructed a gene co-expression network using weighted co-expression network analysis (WGCNA), and conducted clustering analysis. A diagnostic model for diabetic cardiomyopathy was subsequently developed using the least absolute shrinkage and selection operator (LASSO), leading to the identification of six potential biomarkers. This model was validated using additional datasets and cell line experiments.

## Lifestyle modifications: the cornerstone of prevention

At the forefront of preventing cardiovascular complications in T2D is the implementation of lifestyle modifications encouraging to reduce sedentary time and break up sitting time with frequent activity breaks. Regular structured physical activity program stands out as one of the most effective strategies based on aerobic exercise, resistance training and combined exercises. As highlighted by Chen et al. in the published systematic review in this Research Topic, the impact of concurrent aerobic and resistance training enhances vascular health improving endothelial function and reduced arterial stiffness in individuals with T2D even if this kind of training may potentially exacerbate the vascular smooth muscle dysfunction. Further data from well-designed randomized clinical trials will undoubtedly be necessary to investigate this aspect. High-intensity interval training (HIIT) is also effective and has the added benefit of being very time-efficient. Rami et al.‘s research on T2D rats demonstrated that HIIT could significantly mitigate pathological changes in heart tissue, such as hypertrophy, fibrosis, and apoptosis. In fact, HIIT improved molecular indices, including the reduction of B-catenin and c-Myc proteins and an increase in GSK3B and Bcl-2 proteins, suggesting a potential for HIIT to manage cardiovascular complications in diabetes. Furthermore, engaging in at least 150 minutes of moderate-intensity exercise per week can substantially improve cardiovascular health, help control blood sugar levels, and reduce body weight. Exercise enhances insulin sensitivity, lowers blood pressure, and improves lipid profiles, all of which are critical factors in reducing cardiovascular risk (3, 4).

A heart-healthy diet is equally crucial. Diets rich in fruits, vegetables, whole grains, lean proteins, and healthy fats, such as the Mediterranean diet, have been shown to lower cholesterol levels, reduce inflammation, and improve overall cardiovascular health. In the paper of Stelling-Férez et al. the effects of oleanolic acid (OA), a bioactive triterpenoid found in many plants, on endothelial cells from healthy and gestational diabetes-affected pregnancies was investigated. It demonstrated that OA reduces inflammation, decreases monocyte adhesion, enhances angiogenesis, and improves cell migration suggesting its potential on cardiovascular health as well as wound healing. Limiting the intake of processed foods, sugars, and unhealthy fats is essential in managing both diabetes and cardiovascular risk. Additionally, maintaining a healthy weight is vital, as obesity is a significant risk factor for both T2D and CVD. Even modest weight loss can lead to substantial improvements in glycemic control and a reduction in cardiovascular events.

Smoking cessation cannot be overstated in its importance. Smoking exacerbates cardiovascular risk and complicates diabetes management. Smokers with diabetes are at a higher risk of developing heart disease compared to non-smokers, making it imperative to promote smoking cessation programs as part of a comprehensive diabetes management plan.

## Medical management: precision and personalization

To effectively implement personalized prevention strategies, it is crucial to identify specific risk profiles within the T2D population enabling healthcare providers to tailor interventions more precisely, potentially improving patient outcomes and resource allocation in the management of cardiovascular complications associated with T2D. In this Research Topic, Jiménez et al. revealed significant sex and age-related differences in cardiovascular event incidence and presentation among a large cohort of people living with T2D. The findings that men have higher risks for overall CVD, coronary heart disease, and peripheral artery disease, while women face greater risk of heart failure, alongside the observed age-related patterns in disease presentation, underscore the need for personalized approaches in clinical practice.

Effective medical management is fundamental in preventing cardiovascular complications. Regular monitoring and control of blood glucose levels, blood pressure, and cholesterol levels are crucial.

Glycemic control is the cornerstone of diabetes management, with medications such as metformin, sodium-glucose cotransporter 2 inhibitors (SGLT-2i), and Glucagon like peptide-1 (GLP-1) receptor agonists playing a pivotal role. These medications not only lower blood glucose but also provide cardiovascular benefits, including reduced risk of heart failure and improved kidney function. Notably, the network meta-analysis performed by Ghosal and Sinha published in this Research Topic examined the effectiveness of SGLT-2i in reducing cardiovascular death across 13 cardiovascular outcome trials. The analysis confirmed SGLT-2i as a class able to reduce CV death risk; empagliflozin emerged as the most effective agent overall and for people with atherosclerotic CVD, while dapagliflozin showed the best results for subjects affected by heart failure, finally providing valuable insights for tailoring treatment choices in different populations living with T2D and high cardiovascular risk.

Arterial hypertension is a common comorbidity in individuals with T2D and needs to be managed aggressively. In addition to the well-known damages caused by arterial hypertension, it appears that people who simultaneously have T2D and hypertension exhibit significant thalamic changes compared to those without hypertension. This emphasizes the importance of managing hypertension in people living with T2D to prevent further brain damage (Cui et al.). Similarly, dyslipidemia should be addressed with statins or newer lipid-lowering agents (e.g. bempedoic acid, PCSK9 inhibitors) to reduce LDL cholesterol and improve cardiovascular outcomes. Moreover, even though He et al. did not find a causal relationship between serum uric acid levels and heart failure risk in individuals with T2D, suggesting that other factors may play a causal role in heart failure development in this population. In addition, uric acid can activate several pathophysiological mechanisms suggesting a possible causal role of uric acid in the genesis and progression of CVDs. Therefore, it is necessary to investigate and effectively treat hyperuricemia in patients with T2D.

Regular follow-ups with healthcare providers are essential to adjust treatment plans and ensure optimal management of all risk factors. Adherence to prescribed medications is critical, and healthcare providers must emphasize the importance of compliance and regular screenings for early detection of potential complications.

Recently, the investigation of genetic variability has garnered significant interest for its potential to identify subgroups of people living with T2D patients, who possess specific characteristics and may benefit from targeted drug therapies (5).

## Psychosocial and community support: an integral component

Preventing cardiovascular complications in T2D extends beyond medical management and lifestyle changes. Community and psychosocial support are vital in the overall well-being and effective management of diabetes. Support groups, educational programs, and counseling services provide the necessary resources and encouragement for patients to adhere to lifestyle changes and medical regimens. Equally important, the impact of air pollution on CVD risk across the diabetes spectrum is significant, especially in the current context of increasing pollution levels. The review by Bonanni et al. emphasizes the need for healthcare and urban policies to limit exposure to fine particulate matter, as it correlates with elevated blood glucose and HbA1c levels, exacerbating CVD risks in people with T2D. This calls for preventive measures such as using portable air cleaners and other interventions to protect vulnerable populations. Another aspect of great importance to implement is the dissemination of scientific data by researchers and experts in the field, in order to encourage the population to adopt a healthy lifestyle and conscious food consumption.

Addressing the psychosocial aspects of diabetes, such as stress, depression, and anxiety, is equally important. These factors can negatively affect diabetes management and increase cardiovascular risk. Integrating mental health care into diabetes management plans can lead to better outcomes and enhance the quality of life for patients. Healthcare providers should screen for and address mental health issues as part of a comprehensive diabetes care strategy.

## Conclusion: a call to action

Preventing cardiovascular complications in T2D requires a multifaceted approach. Lifestyle modifications, effective medical management, and robust community and psychosocial support are all critical components. By addressing these areas comprehensively, we can significantly reduce the burden of CVD in individuals with T2D, improve their quality of life, and extend their life expectancy.

Healthcare professionals, policymakers, and communities must collaborate to implement strategies that promote heart health and diabetes management. This includes creating supportive environments that encourage healthy lifestyles, providing access to effective medical treatments, and offering psychosocial support. By taking a proactive and holistic approach, we can make significant strides in preventing cardiovascular complications in T2D and ensuring that patients receive the comprehensive care they need to thrive.
